# Global Suitability Areas of Two Rice Pests, *Nilaparvata lugens* and *Sogatella furcifera*, and Their Management Implications

**DOI:** 10.1002/ece3.72637

**Published:** 2025-12-29

**Authors:** Zhengxue Zhao, Xueli Feng, Yingjian Wang, Yubo Zhang

**Affiliations:** ^1^ College of Agriculture Anshun University Anshun China

**Keywords:** climate change, Maxent, pest management, planthopper, rice pest, suitability areas

## Abstract

Rice is a staple food crop for over half of the global population; however, it is being increasingly threatened by the planthopper species *Nilaparvata lugens* and *Sogatella furcifera* (Hemiptera: Delphacidae). Effective management of these pests is crucial for maintaining stable rice production, which requires an understanding of their geographical distribution. Unfortunately, previous studies have generally focused on limited geographic and environmental scopes. Therefore, in this study, a Maxent model was employed to predict the global suitability areas of both pests under current and future conditions (2050s and 2070s) using occurrence records and a broad range of environmental variables. Model results revealed that precipitation‐ and temperature‐related factors are the primary drivers of both pests' distribution. Asia, particularly China, was predicted to remain the most suitable region for them now and in the future, underscoring the urgency of reinforcing pest management efforts in these regions. Owing to climate change, the suitability areas for the two pests are expected to expand and shift northward, with the most pronounced increase under the 2070s SSP585 scenario. Furthermore, this expansion of high suitability areas suggests a growing risk of outbreaks. By identifying regions at risk of infestation and quantifying the extent of suitable habitat expansion, our findings provide insights for developing targeted, region‐specific pest management strategies for *N. lugens* and *S. furcifera* , to minimize food and economic losses.

## Graphical abstract

Suitable areas for both pests are projected to expand and shift northward by future. Asia remains the most affected region under all future climate scenarios. Findings support targeted, climate‐informed pest control strategies worldwide.

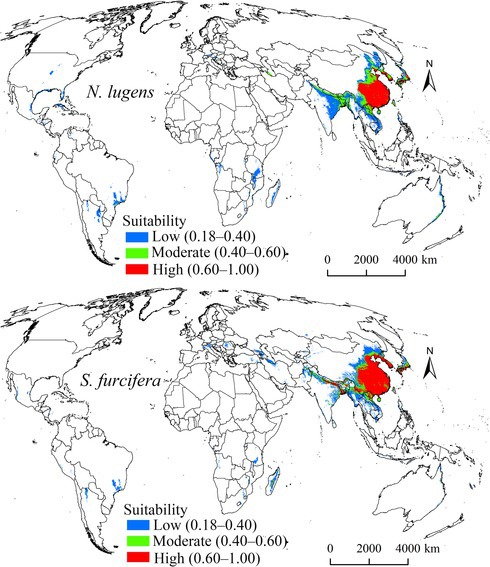

## 1 Introduction

Rice ( *Oryza sativa* L.) is the third most cultivated crop worldwide and has become a major food source for over 50% of the global population (Liu et al. 2019). Rice production varies across regions but is mainly concentrated in Asia, accounting for 89.6% of global output (FAO 2025). The top 10 rice‐producing countries are in Asia, including China, India, and Bangladesh (FAO 2025). Food security and economic stability are closely linked to rice production (Zhang et al. 2015; Shi et al. 2023); thus, the stability and sustainability of rice production are vital to human society.

*Nilaparvata lugens* and *Sogatella furcifera* are two destructive rice planthoppers. They were originally restricted to Asia but have now established populations in other regions, such as Africa. Moreover, they damage rice crops by sucking phloem sap, laying eggs, and transmitting viruses (Zhou et al. 2008, 2018), with the affected area and economic losses being substantial. For example, in Asia, *N. lugens* causes annual losses exceeding 300 million US dollars (Min et al. 2014); during major outbreaks, it has damaged up to 20 million hectares of rice per year in China (Hu et al. 2011, 2014; Lu et al. 2017). Additionally, *S. furcifera* has severely damaged 26,700 ha of rice fields, causing a loss of 190,000 tons of grain (Gui et al. 2008). Unfortunately, effective management of *N. lugens* and *S. furcifera* has become increasingly challenging despite tremendous efforts. This is mainly owing to their adaptability, long‐distance migration, and increased pesticide resistance (Hu et al. 2014; Wang et al. 2019).

Accurate management of *N. lugens* and *S. furcifera* requires a clear understanding of their geographic distribution. Their occurrences are closely related to various environmental conditions, especially climate. Unfortunately, global climate is undergoing notable changes and is expected to expand the geographic distribution of these two pests (Hu, Liu, et al. 2015; Hu, Fu, et al. 2015). This expansion will lead to the emergence of new infestation areas and increase the likelihood of outbreaks (Hu et al. 2011; Hu, Fu, et al. 2015), thereby posing substantial challenges to control efforts. Therefore, predicting changes in the distribution of these pests under future climate conditions is imperative. Such predictions are essential for implementing timely and effective pest management strategies to reduce rice losses and safeguard food security.

Species distribution models are especially valuable for predicting species ranges by linking occurrence data with environmental variables. In agriculture, these models are widely used to project pest suitability under future climate scenarios (Zhang et al. 2023; Wei et al. 2024; Zhao et al. 2024a, 2024b), revealing spatial and temporal dynamics. Such insights can identify risk areas and guide the development of more efficient pest control strategies (Sanyal et al. 2025; Wang et al. 2025). Several studies have applied species distribution models to *N. lugens* and *S. furcifera* (Hu, Liu, et al. 2015; Guru‐Pirasanna‐Pandi et al. 2021; Xu et al. 2021; Hong et al. 2024; Xiu et al. 2024; Surmaini et al. 2024). However, these studies often used default settings without calibration and relied on outdated climate projections. Moreover, most species distribution models have incorporated only bioclimatic variables, overlooking the roles of rice, wind speed, and topographic features. Rice is the primary host plant for both pests, and wind speed is a key driver of their long‐distance migration (Lu et al. 2017; Wu et al. 2022); moreover, topography strongly influences their range through local environmental conditions (Liu et al. 2013; Hu, Liu, et al. 2015). Therefore, these environmental variables are critical to the distribution of the two pests, and their inclusion can yield more accurate predictions. Furthermore, studies have focused mainly on regional scales (e.g., Asia, China, Indonesia, and India) and have not developed global‐scale models. Therefore, the global suitability areas of *N. lugens* and *S. furcifera* and their projected changes remain poorly understood. To address the limitations in predicting the distributions of *N. lugens* and *S. furcifera* , we employed Maxent, the most widely used species distribution model, to estimate the global suitability areas of these pests based on global occurrence records and a comprehensive set of environmental variables. In particular, this study addresses four key questions: (1) What are the main environmental variables determining the distributions of the two pests? (2) Where are the current and future suitability areas? (3) How will these areas shift under future climate change scenarios? (4) How can this knowledge guide pest management for the two pests?

## 2 Materials and Methods

### 2.1 Species Occurrence Records

**FIGURE 1:**
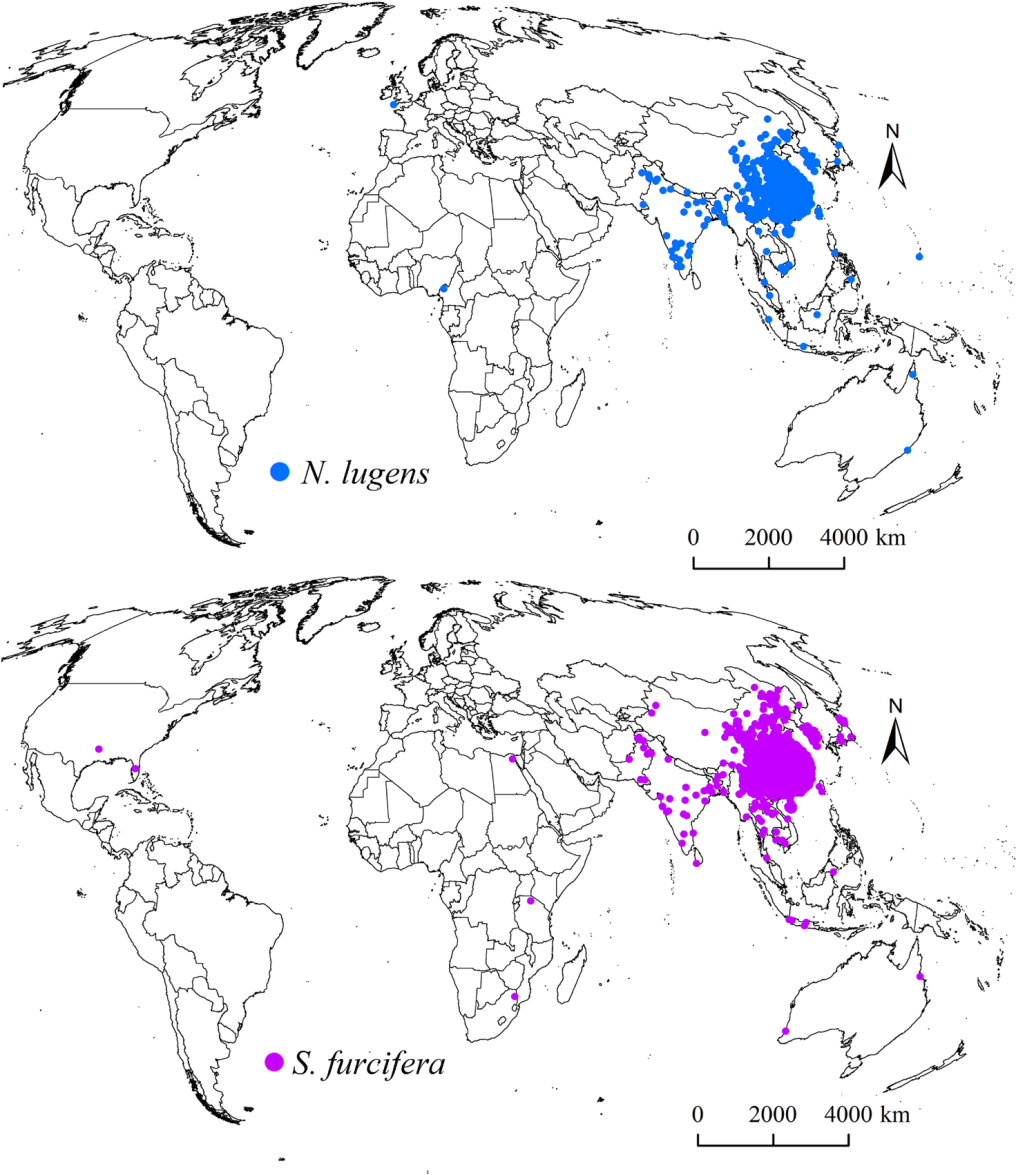
Occurrence of *N. lugens* and *S. furcifera* worldwide.

In total, 847 and 1284 occurrence records for *N. lugens* and *S. furcifera* , respectively, were used to construct a Maxent model (Figure 1). These data were originally sourced from the literature, specimen records from Anshun and Guizhou Universities, and the Global Biodiversity Information Facility (GBIF 2025; https://www.gbif.org/). Occurrence records lacking geographic coordinates were georeferenced in Google Earth based on locality descriptions. GBIF records were cleaned by removing entries with common biological collection errors and coordinate uncertainty (> 5 km) using the CoordinateCleaner Package (Zizka et al. 2019). Because sampling bias in occurrence data can markedly affect model performance, records were spatially thinned at a 5‐km distance using the spThin package (Aiello‐Lammens et al. 2015).

### 2.2 Environmental Variable Selection

Current environmental data included 19 bioclimatic variables (BIO1–BIO19) for 1970–2000, 3 topographic features (elevation, slope, and terrain ruggedness), wind speed, and rice‐growing area. Bioclimatic variables were divided into temperature‐related (BIO1–BIO11) and precipitation‐related (BIO12–BIO19) groups. These variables were selected because they capture climatic constraints, local environmental conditions, long‐distance migration, and host plant availability that determine the distribution of *N. lugens* and *S. furcifera* . To minimize collinearity, Pearson correlation was used to select variables. Values were extracted from occurrence records, and pairwise Pearson correlation coefficients (*r*) were calculated using the sjPlot package (Lüdecke 2024). When two environmental variables had *|r|* > 0.75, one was retained. Subsequently, the variance inflation factor (VIF) for retained variables was checked using the usdm package (Naimi et al. 2014), confirming that all VIF values were below 5. Finally, 11 and 9 environmental variables were retained for *N. lugens* and *S. furcifera* , respectively.

Future bioclimatic variables were obtained from the Coupled Model Intercomparison Project Phase 6 for 2041–2060 (2050s) and 2061–2080 (2070s) under Shared Socioeconomic Pathway (SSP) 126, 245, and 585. To reduce uncertainty associated with using a single climate model, future bioclimatic variables were derived from the mean of five climate models: BCC‐CSM2‐MR, HadGEM3‐GC31‐LL, IPSL‐CM6A‐LR, MIROC6, and MPI‐ESM1‐2‐HR. These climate models have been widely used to predict the future distributions of species (Zhao et al. 2024a; Zhang et al. 2024; Arslan et al. 2025; Zhu et al. 2025; Luo et al. 2025). Current terrain ruggedness, wind speed, and rice‐growing area data were used for future distribution predictions.

Bioclimatic variable, elevation, and wind speed data were sourced from WorldClim (https://www.worldclim.org) at a 2.5‐min spatial resolution. Slope and terrain ruggedness were derived from elevation data using the terra package (Hijmans 2025). Rice‐growing area data at a 1‐km spatial resolution were obtained from the SPAM 2020 v1.0 Global dataset (International Food Policy Research Institute 2024) and resampled to the 2.5‐min spatial resolution.

### 2.3 Maxent Model Construction

In the Maxent model, feature classes (FCs) and the regularization multiplier (RM) are parameters that control model complexity (Muscarella et al. 2014; Radosavljevic and Anderson 2014) and markedly influence prediction results. FCs are of five types: linear (L), quadratic (Q), hinge (H), product (P), and threshold (T). RM represents a penalty for model complexity, preventing overfitting. The best FC–RM combination was selected based on the lowest corrected Akaike information criterion using Genetic Algorithm implemented in the SDMtune package (Vignali et al. 2020). In total, 2400 hyperparameter combinations were used across 10 FC combinations (“L,” “H,” “LQ,” “LQH,” “LQP,” “HPT,” “QHP,” “LQHP,” “QHPT,” and “LQHPT”) and RM values from 0.10 to 4.88 (in 0.02 increments). The optimal settings were (i) LQHPT for FC and 2.7 for RM for *N. lugens* and (ii) LQP for FC and 0.24 for RM for *S. furcifera* . Model construction used 10,000 background points, 5 replicates with cross‐validation, and loglog output via the dismo package (Hijmans et al. 2023). The jackknife test was used to evaluate the relative importance of each environmental variable. Model performance was assessed using the area under the receiver operating characteristic curve (AUC), a threshold‐independent evaluation metric. AUC values ranged from 0 to 1, with values greater than 0.9 indicating outstanding performance (Peterson et al. 2011; Bogawski et al. 2019; Gao et al. 2021).

Extrapolation occurs when a model predicts under new environmental conditions in a new geographic area, making such predictions require caution. To examine the risk of extrapolation, this study used multivariate environmental similarity surface (MESS) analysis (Elith et al. 2010), implemented in the dismo package (Hijmans et al. 2023). This analysis calculates similarity by comparing environmental conditions represented in occurrence records with environmental rasters of the model‐projected area to produce maps of environmental similarity. A similarity value of < 0 indicates a risk of extrapolation, whereas values of ≥ 0 indicate no extrapolation but rather interpolation (Sutton and Martin 2022).

### 2.4 Prediction Visualization

The maximum training sensitivity plus specificity threshold was used to classify predicted raster values from 0 to 1 as suitable or unsuitable. Areas above the threshold were considered suitable, whereas those below were deemed unsuitable. A threshold of 0.18 was applied for *N. lugens* and *S. furcifera* . Suitability areas were further subdivided into low (0.18–0.40), moderate (0.40–0.60), and high (0.60–1.00) suitability (Zhao, Yang, Long, et al. 2024; Feng et al. 2025). This gradient provides a clear view of habitat suitability and aids in pest management planning.

To explore spatial changes in suitability areas under future climate conditions, expansion, contraction, and stable zones relative to the current period were identified based on binary prediction results (low, moderate, and high suitability). This analysis was conducted using the “Distribution changes between binary SDMs” tool in SDMtoolbox for ArcGIS 10.7 (Brown 2014). The three zones of spatial distribution patterns and their areas (km^2^) were obtained automatically.

## 3 Results

### 3.1 AUC Values and Variable Importance

**FIGURE 2:**
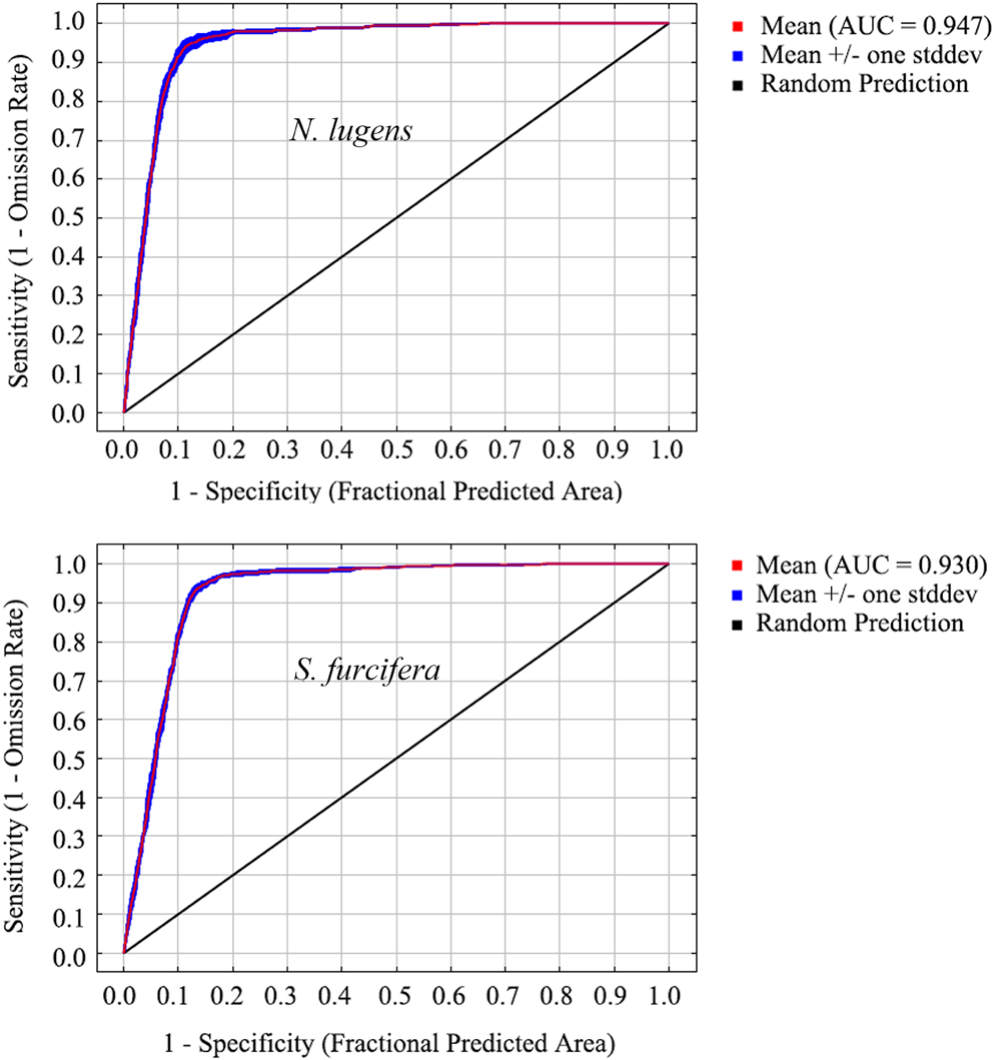
AUC value for *N. lugens* and *S. furcifera* .

The mean (±standard deviation) test AUC of five replicates was 0.947 (±0.003) and 0.930 (±0.002) for *N. lugens* and *S. furcifera* , respectively (Figure 2), indicating outstanding model performance and strong reliability in predicting suitable areas.

### 3.2 Variable Importance

**FIGURE 3:**
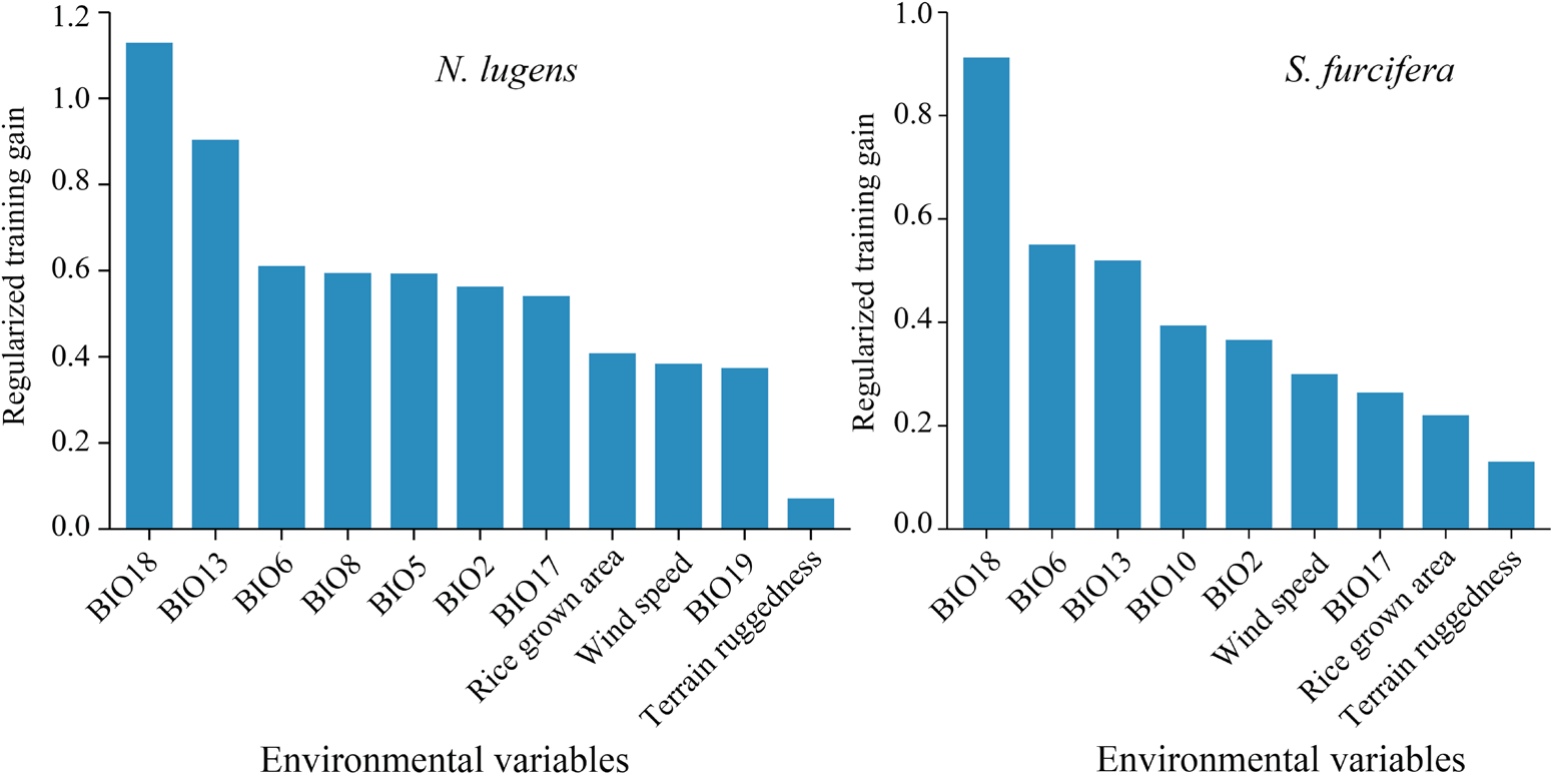
Importance of environmental variables for *N. lugens* and *S. furcifera* occurrence.

**FIGURE 4:**
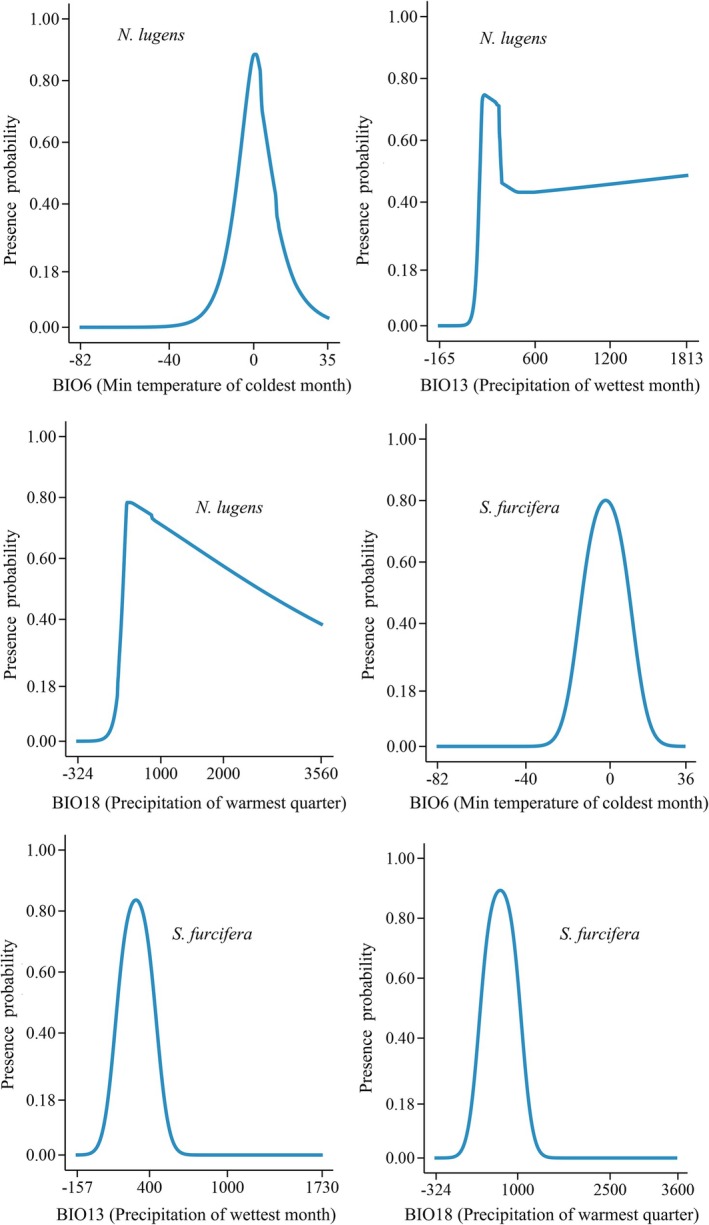
Response curves showing the relationships between presence probability and the key environmental variables for *N. lugens* and *S. furcifera* .

The jackknife test revealed that BIO18 (precipitation of warmest quarter) was the most important environmental variable influencing the distribution of *N. lugens* , followed by BIO13 (precipitation of wettest month) and BIO6 (min temperature of coldest month) (Figure 3). Other important variables, in descending order (Figure 3), included BIO8 (mean temperature of wettest quarter), BIO5 (max temperature of warmest month), BIO2 (mean diurnal range), and BIO17 (precipitation of driest quarter). Rice‐growing area, wind speed, BIO19 (precipitation of coldest quarter), and terrain ruggedness had relatively low importance, ranking in the bottom four variables (Figure 3). For *S. furcifera* , BIO18 (precipitation of warmest quarter) also had the highest influence, with BIO6 (min temperature of coldest month) and BIO13 (precipitation of wettest month) showing substantial contributions (Figure 3). BIO10 (mean temperature of warmest quarter), BIO2 (mean diurnal range), wind speed, BIO17 (precipitation of driest quarter), rice‐growing area, and terrain ruggedness followed sequentially in importance (Figure 3). Overall, precipitation‐ and temperature‐related variables were the primary factors affecting the distributions of *N. lugens* and *S. furcifera* . The response curves further demonstrated that the probability of presence for both pest species initially increased and then declined with rising BIO6 (min temperature of coldest month), BIO13 (precipitation of wettest month), and BIO18 (precipitation of warmest quarter) values, displaying unimodal relationships (Figure 4). The ranges of the three main variables for the presence probability and high‐presence probability of *N. lugens* and *S. furcifera* varied (Table 1).

**TABLE 1:** Ranges of the three main variables for presence probability and high‐presence probability of *N. lugens* and *S. furcifera* .

Species	Variable	Presence probability (0.18–1.00)	High‐presence probability (0.60–1.00)
*N. lugens*	BIO6	−15.01°C–17.40°C	−5.68°C–6.20°C
	BIO13	129.86–1812.8 mm	165.45–317.73 mm
	BIO18	313.53–3562.9 mm	418.47–1817.72 mm
*S. furcifera*	BIO6	−19.56°C–15.30°C	−11.16°C–6.90°C
	BIO13	78.25–513.94 mm	175.90–414.41 mm
	BIO18	64.78–1370.74 mm	437.91–997.61 mm

### 3.3 Patterns and Changes of Suitable Areas

**FIGURE 5:**
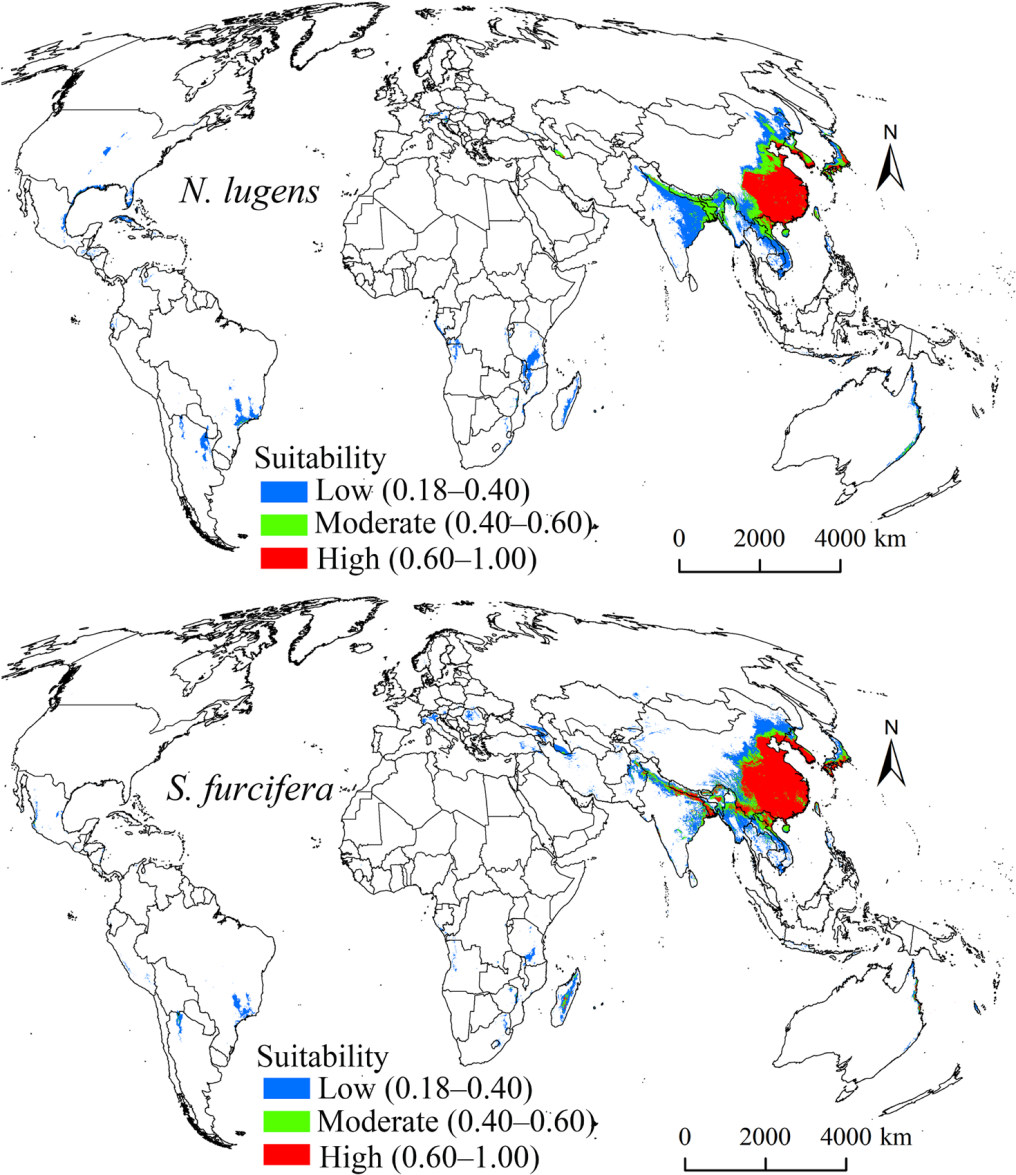
Global distribution of current suitability areas for *N. lugens* and *S. furcifera* .

The Maxent model predicted a total current suitability area of 8.13 × 10^6^ km^2^ for *N. lugens* (Table 2), predominantly in Asia (Figure 5), especially China, Vietnam, Laos, India, Bangladesh, Nepal, South Korea, North Korea, and Japan. The extent and distribution of suitability levels varied (Figure 5), with high suitability areas concentrated in China, South Korea, North Korea, and Japan. Moderate suitability areas were mostly found in China, South Korea, Bangladesh, Nepal, and Japan (Figure 5), whereas low suitability areas were mostly found in China, India, Vietnam, and Japan. Outside Asia, only low suitability areas were detected (Figure 5).

**TABLE 2:** Projected suitability areas (km^2^) for *N. lugens* , with areas categorized by suitability.

Suitability	Current	2050s SSP126	2050s SSP245	2050s SSP585	2070s SSP126	2070s SSP245	2070s SSP585
Total	8.13 × 10^6^	9.52 × 10^6^ (+17.09%)	9.67 × 10^6^ (+18.94%)	1.00 × 10^7^ (+23.00%)	9.53 × 10^6^ (+17.22%)	1.01 × 10^7^ (+24.23%)	1.08 × 10^7^ (+32.84%)
Low	4.03 × 10^6^	4.72 × 10^6^ (+17.12%)	4.62 × 10^6^ (+14.64%)	4.85 × 10^6^ (+20.34%)	4.70 × 10^6^ (+16.62%)	4.97 × 10^6^ (+23.32%)	5.28 × 10^6^ (+31.01%)
Moderate	1.79 × 10^6^	1.60 × 10^6^ (−10.61%)	1.71 × 10^6^ (−4.46%)	1.73 × 10^6^ (−3.35%)	1.60 × 10^6^ (−10.61%)	1.74 × 10^6^ (−2.79%)	1.91 × 10^6^ (+6.70%)
High	2.32 × 10^6^	3.21 × 10^6^ (+38.36%)	3.34 × 10^6^ (+43.96%)	3.45 × 10^6^ (+48.70%)	3.22 × 10^6^ (+38.79%)	3.36 × 10^6^ (+44.82%)	3.65 × 10^6^ (+57.32%)

*S. furcifera* 's predicted current suitability areas were also concentrated in several Asian countries (Figure 5), namely China, Vietnam, Laos, Burma, India, Bangladesh, Nepal, South Korea, North Korea, and Japan. Compared with those for *N. lugens* , the suitability areas for *S. furcifera* were smaller in Africa, North America, South America, and Australia but larger in Europe (Figure 5). High suitability areas for *S. furcifera* were mainly distributed in China, India, Bangladesh, Nepal, South Korea, North Korea, and Japan (Figure 5). Moderate suitability covered the smallest area, whereas low suitability was the most widespread (Table 3).

**TABLE 3:** Projected suitability areas (km^2^) for *S. furcifera* , with areas categorized by suitability.

Suitability	Current	2050s SSP126	2050s SSP245	2050s SSP585	2070s SSP126	2070s SSP245	2070s SSP585
Total	8.19 × 10^6^	9.71 × 10^6^ (+18.55%)	1.00 × 10^7^ (+22.10%)	1.05 × 10^7^ (+28.20%)	9.69 × 10^6^ (+18.31%)	1.05 × 10^7^ (+28.20%)	1.29 × 10^7^ (+57.50%)
Low	3.43 × 10^6^	4.36 × 10^6^ (+27.11%)	4.53 × 10^6^ (+32.06%)	4.79 × 10^6^ (+39.65%)	4.30 × 10^6^ (+25.36%)	4.82 × 10^6^ (+40.52%)	6.57 × 10^6^ (+91.54%)
Moderate	1.39 × 10^6^	1.58 × 10^6^ (+13.66%)	1.60 × 10^6^ (+15.10%)	1.71 × 10^6^ (+23.02%)	1.58 × 10^6^ (+13.66%)	1.74 × 10^6^ (+25.17%)	1.80 × 10^6^ (+29.49%)
High	3.36 × 10^6^	3.76 × 10^6^ (+11.90%)	3.89 × 10^6^ (+15.77%)	4.01 × 10^6^ (+19.34%)	3.80 × 10^6^ (+13.09%)	3.98 × 10^6^ (+18.45%)	4.51 × 10^6^ (+34.22%)

**FIGURE 6:**
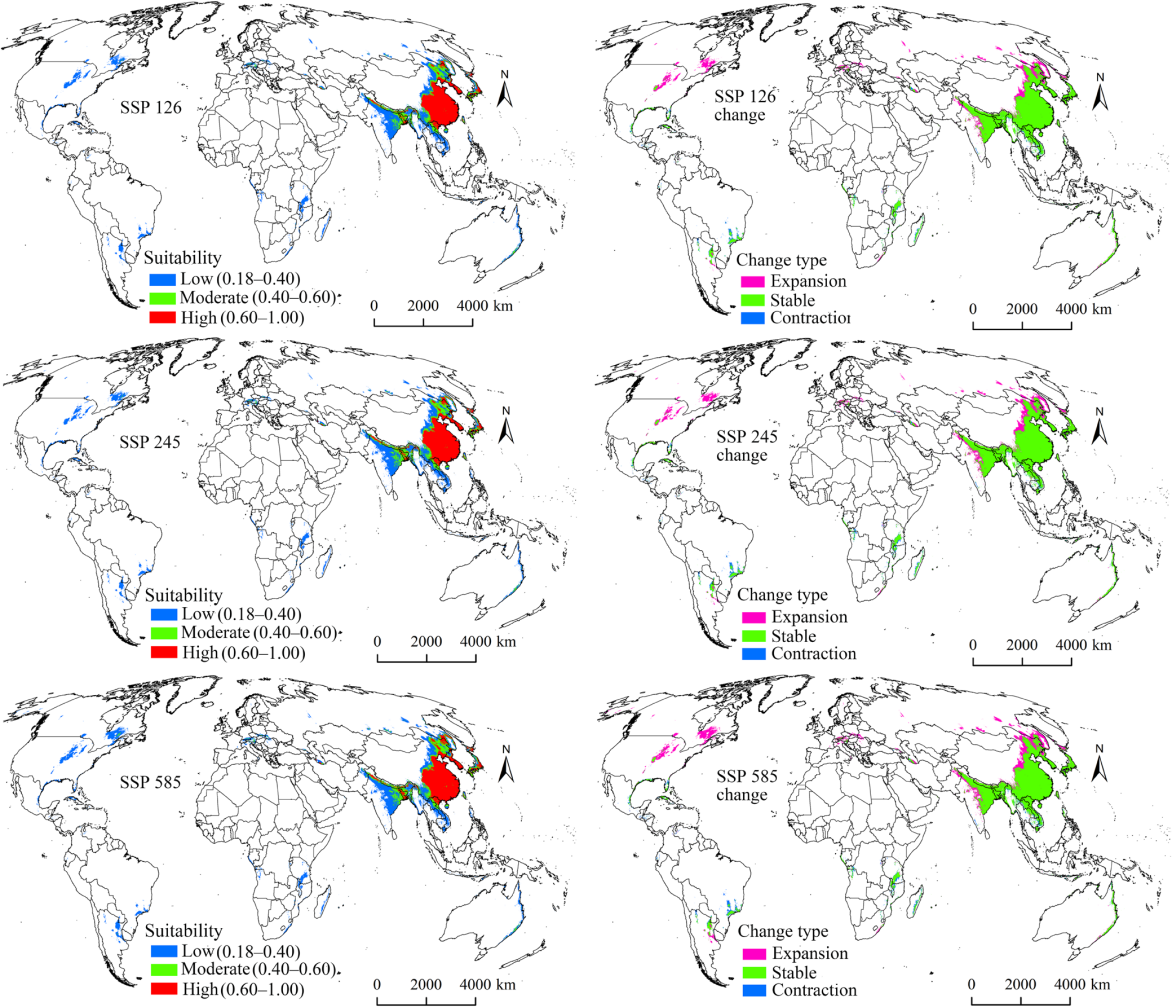
Suitability areas and changes for *N. lugens* in the 2050s.

**FIGURE 7:**
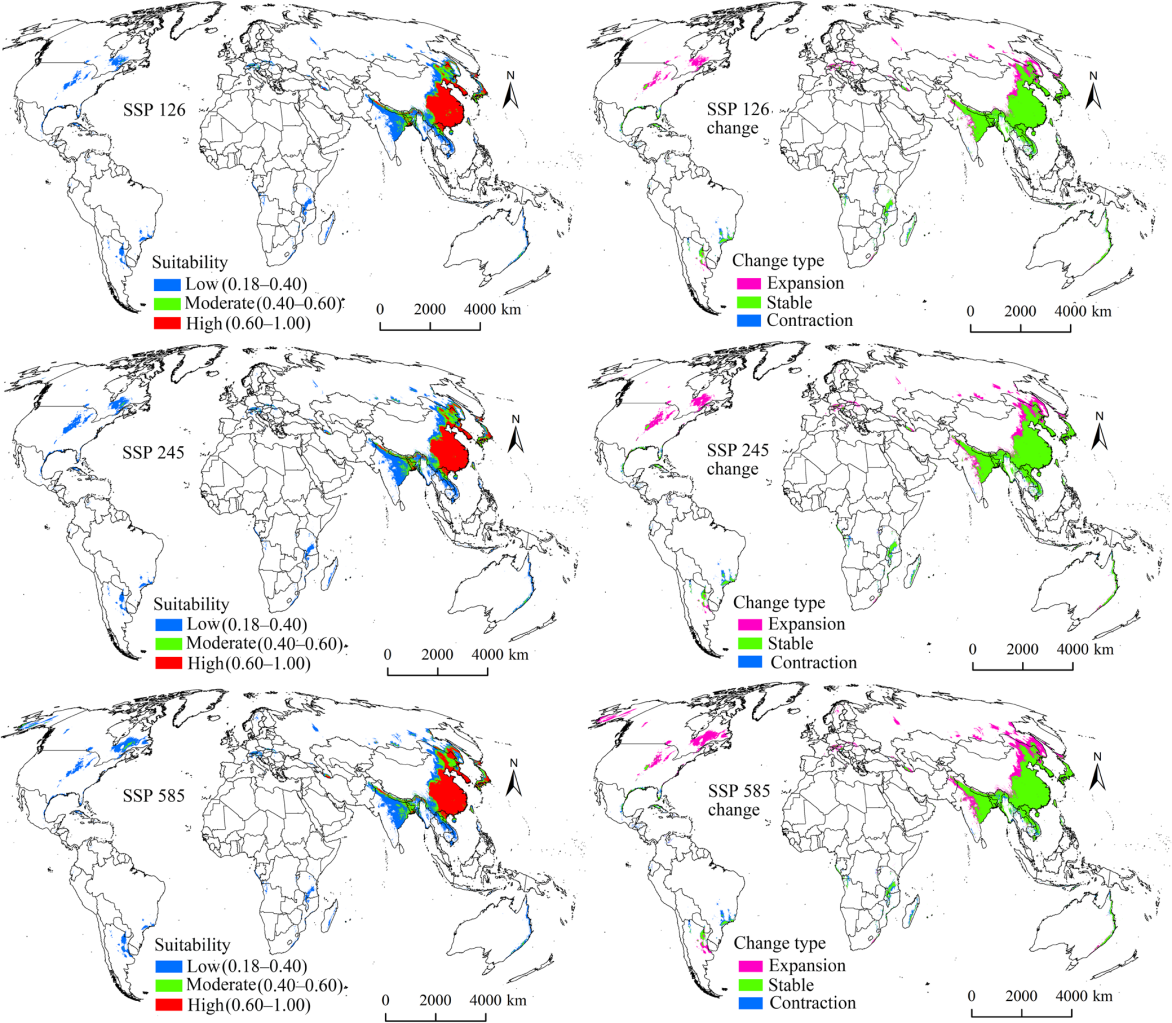
Suitability areas and changes for *N. lugens* in the 2070s.

**FIGURE 8:**
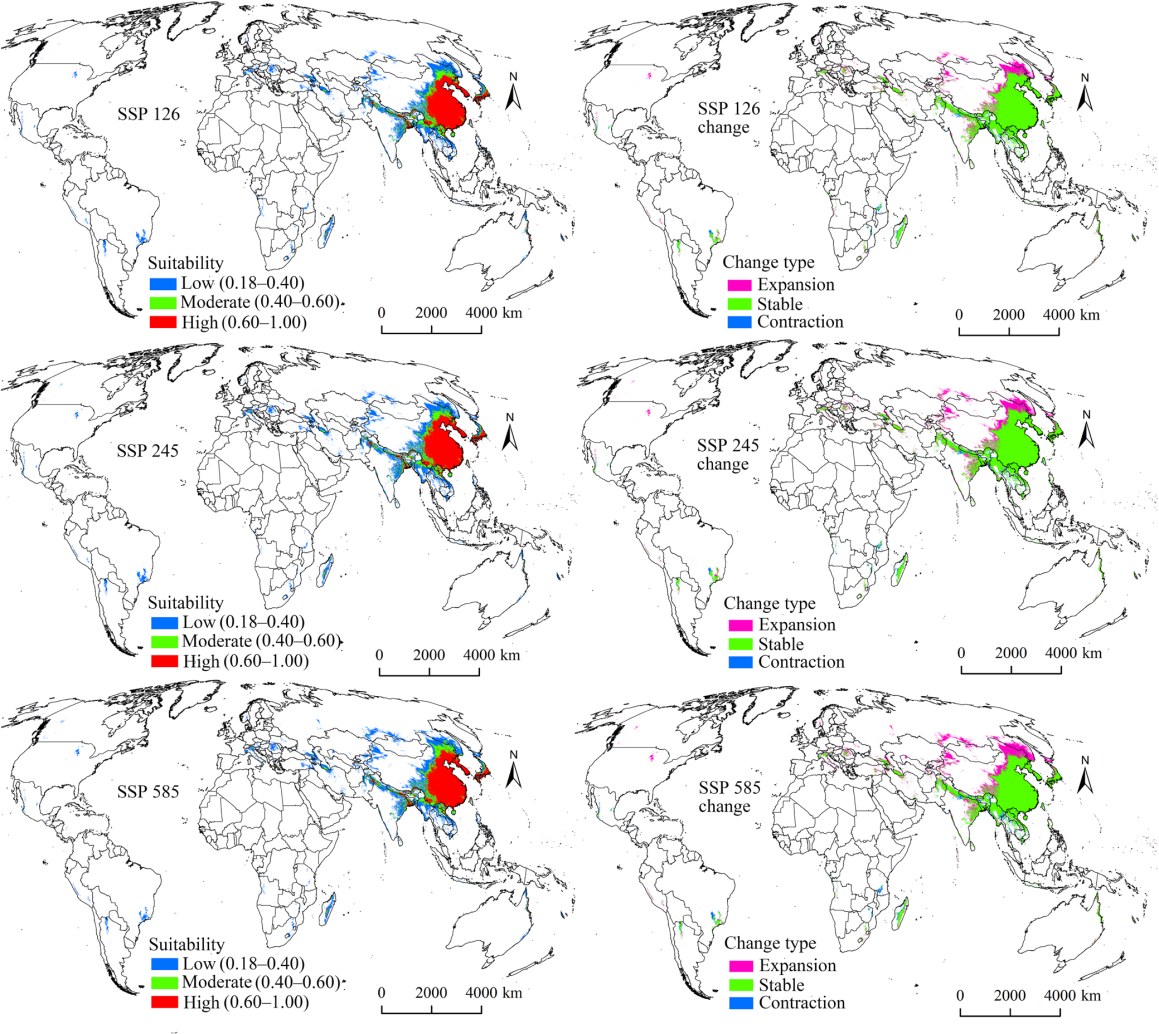
Suitability areas and changes for *S. furcifera* in the 2050s.

**FIGURE 9:**
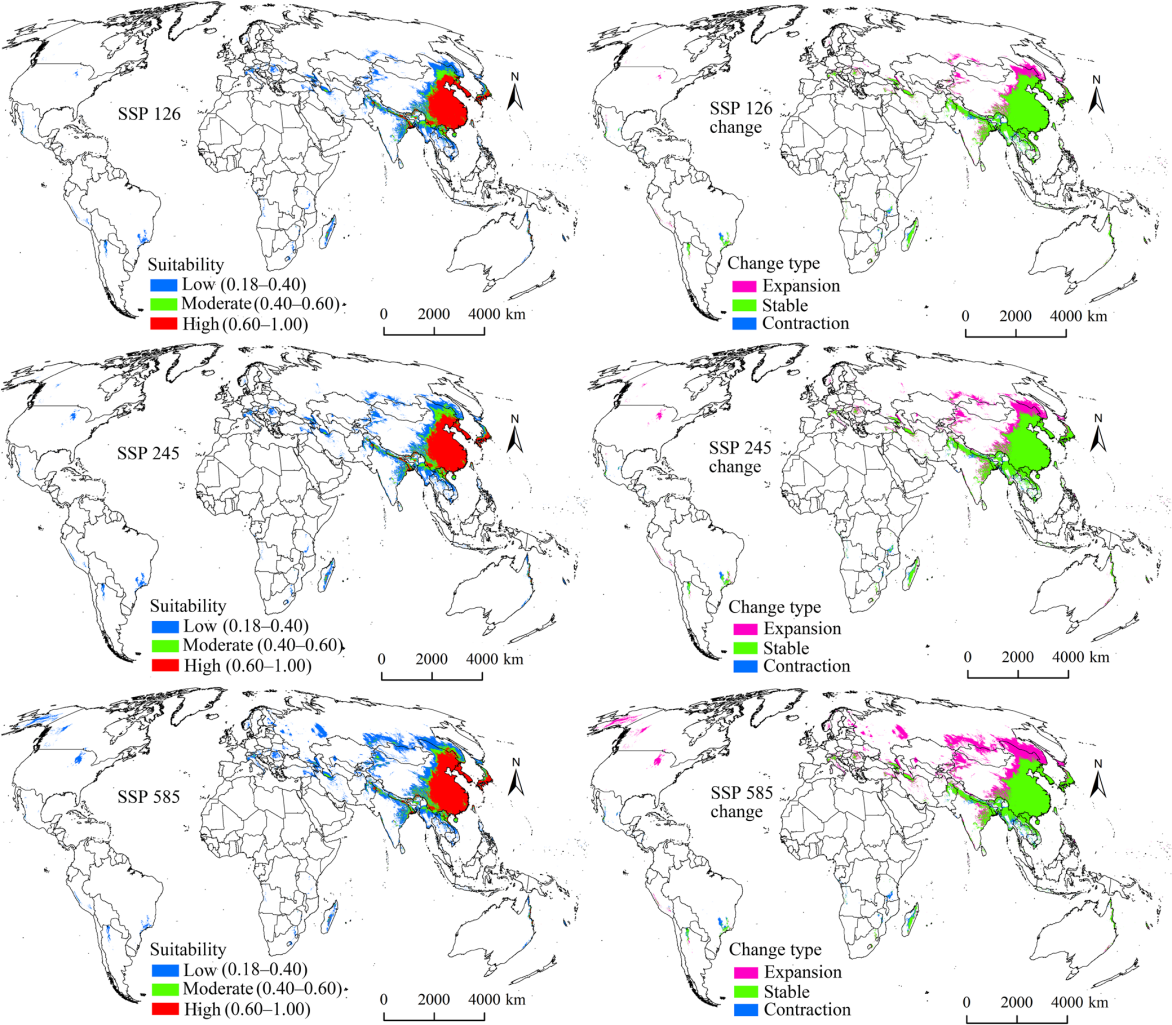
Suitability areas and changes for *S. furcifera* in the 2070s.

Under all future climate scenarios, suitability areas for both pests were projected to expand beyond current ranges (Figures 6, 7, 8, 9), confirming that climate change will drive distribution growth. The extent of increase in suitability areas varied across future climate scenarios, ranging from 17.09% to 32.84% for *N. lugens* and from 18.31% to 57.50% for *S. furcifera* (Tables 2 and 3), whereas core suitability areas remained largely consistent with the current distribution in Asia (Figures 6, 7, 8, 9). For *N. lugens* , both high and low suitability areas were projected to increase, whereas moderate suitability was expected to decline compared with the present period, except for the SSP585 scenario in 2070s (Table 2). In contrast, *S. furcifera* was predicted to experience increases of varying magnitudes across all suitability levels (Table 3). Total and categorized suitability areas for both species were expected to peak by the 2070s under the SSP585 scenario (Tables 2 and 3).

The expansion, stable, and contraction areas representing all suitability levels were identified (Tables 4 and 5). Expansion areas for both pests under all future climate scenarios were primarily located north of their current ranges, particularly in North America, Europe, and northern Asia (Figures 6, 7, 8, 9), indicating a northward shift driven by climate change. Expansion is projected to be most pronounced during the 2070s under the SSP585 scenario (Figures 7 and 9). Additionally, *S. furcifera* was expected to have larger expansion zones compared with those of *N. lugens* under each scenario (Tables 4 and 5). Predicted stable areas for both pest species were the largest category (Tables 4 and 5), concentrated in Asia, especially China, Vietnam, Laos, India, South Korea, North Korea, and Japan (Figures 6, 7, 8, 9). In contrast, contraction areas were limited (Tables 4 and 5), mainly distributed in Brazil and Argentina (Figures 6, 7, 8, 9).

**TABLE 4:** Changes in suitability areas (km^2^) for *N. lugens* under future climate scenarios relative to the current period.

Change type	2050s SSP126	2050s SSP245	2050s SSP585	2070s SSP126	2070s SSP245	2070s SSP585
Expansion	1.87 × 10^6^	2.05 × 10^6^	2.48 × 10^6^	1.91 × 10^6^	2.55 × 10^6^	3.53 × 10^6^
Stable	7.76 × 10^6^	7.72 × 10^6^	7.65 × 10^6^	7.72 × 10^6^	7.63 × 10^6^	7.42 × 10^6^
Contraction	4.62 × 10^5^	4.98 × 10^5^	5.70 × 10^5^	4.97 × 10^5^	5.89 × 10^5^	7.98 × 10^5^

**TABLE 5:** Changes in suitability areas (km^2^) for *S. furcifera* under future climate scenarios relative to the current period.

Change type	2050s SSP126	2050s SSP245	2050s SSP585	2070s SSP126	2070s SSP245	2070s SSP585
Expansion	1.88 × 10^6^	2.25 × 10^6^	2.84 × 10^6^	1.94 × 10^6^	2.84 × 10^6^	5.42 × 10^6^
Stable	7.92 × 10^6^	7.88 × 10^6^	7.77 × 10^6^	7.84 × 10^6^	7.82 × 10^6^	7.59 × 10^6^
Contraction	3.45 × 10^5^	3.87 × 10^5^	4.98 × 10^5^	4.25 × 10^5^	4.54 × 10^5^	6.80 × 10^5^

**FIGURE 10:**
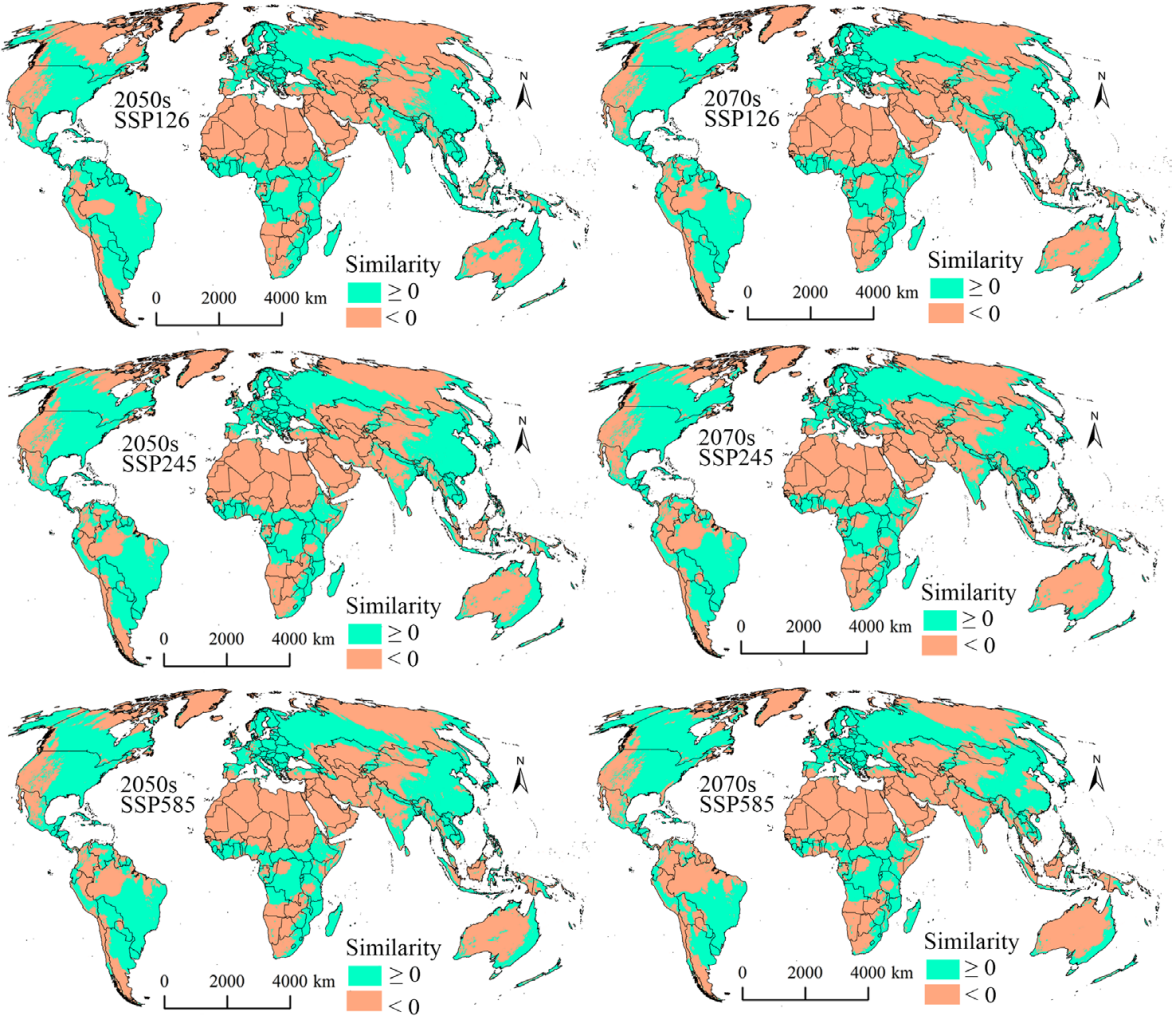
MESS analysis results for *N. lugens* in the 2050s and 2070s.

**FIGURE 11:**
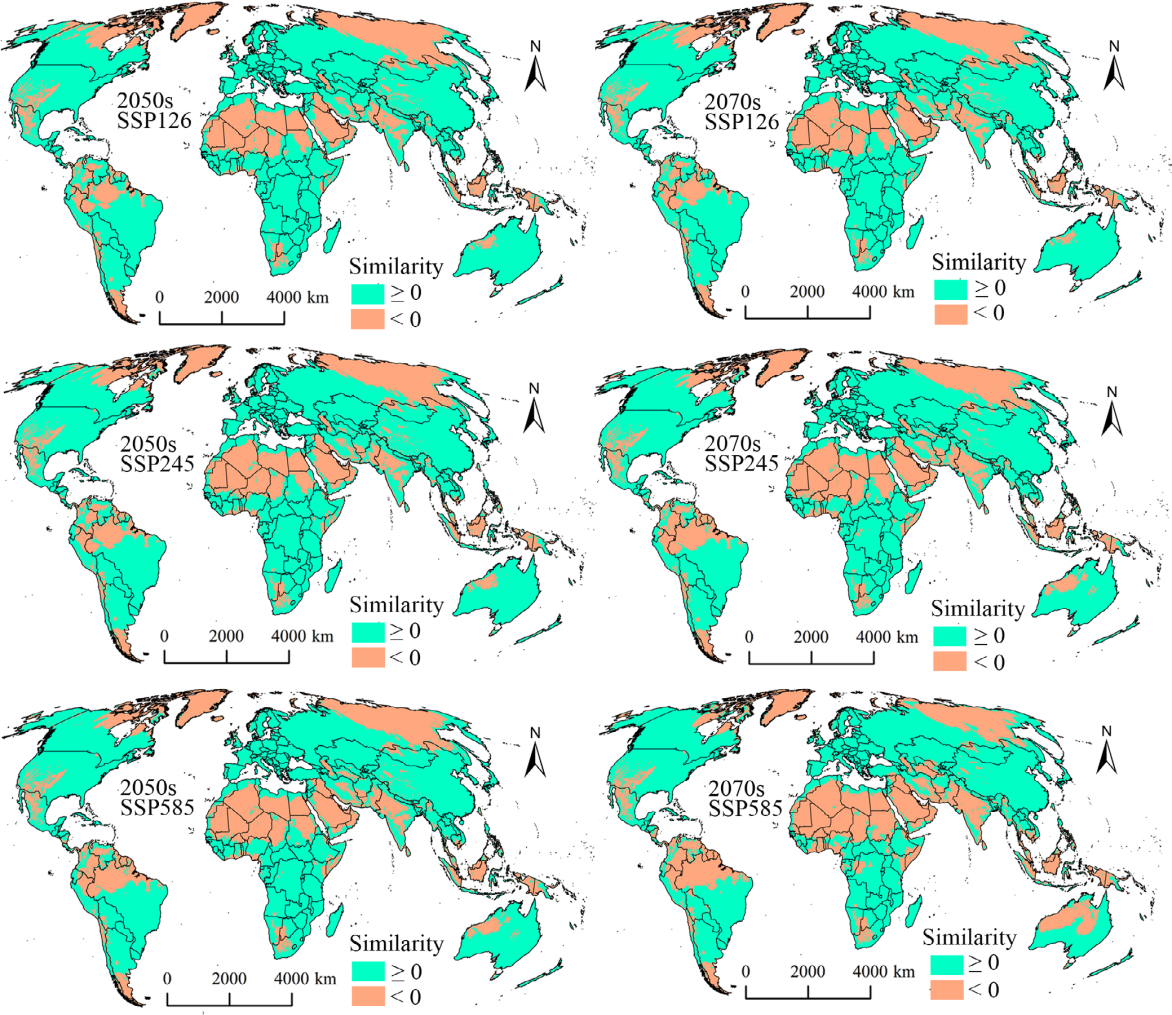
MESS analysis results for *S. furcifera* in the 2050s and 2070s.

Comparison of the model predictions with the MESS analysis results showed that only a considerably small portion of the suitable areas fell into extrapolation risk zones (similarity values < 0) for the two pests (Figures 10 and 11). For *N. lugens* , a small fraction of the predicted suitable areas in eastern India and eastern China fell within such zones, most notably under the 2070s SSP585 climate scenario (Figure 10). For *S. furcifera* , extrapolation was largely restricted to eastern India (Figure 11). Overall, the extrapolation risk of the developed models for the two pests was minimal, and the prediction results demonstrated high reliability.

## 4 Discussion

The model developed in this study revealed that precipitation‐ and temperature‐related variables are the primary environmental factors affecting the distributions of *N. lugens* and *S. furcifera* , exhibiting unimodal relationships. Precipitation during the warmest quarter and wettest month typically coincides with rice‐growing periods. Within a suitable range, increased precipitation can create a humid microclimate in rice fields that favors egg hatching and nymph development, thereby promoting the population establishment of both pests. However, excessive precipitation can lead to water accumulation, potentially submerging eggs or individuals on rice plants and reducing population size. In addition to these direct effects, precipitation may also influence *N. lugens* and *S. furcifera* distribution indirectly via rice, serving as a host. Nonetheless, the established model indicated that the influence of rice‐growing area is weaker than that of precipitation variables (Figure 3), suggesting that the direct effects of precipitation play a more important role than indirect effects mediated through rice.

Low winter temperatures are also critical in determining the distribution of *N. lugens* and *S. furcifera* (Heong et al. 2015; Hu, Fu, et al. 2015). These pests can overwinter only in tropical regions with considerably high temperatures, whereas populations in temperate regions are migratory, originating from tropical areas (Matsumoto et al. 2013; Hu et al. 2013, 2018; Matsumura et al. 2018). Therefore, areas with predicted presence probabilities below 0°C in the coldest month are likely to be migration zones, whereas those with predicted presence probabilities above 0°C are likely to be overwintering zones. The results of this study also showed that high and low winter temperatures are unsuitable for *N. lugens* and *S. furcifera* survival. This may be due to high‐density competition in tropical overwintering populations, which reduces individual survival chances, and colder conditions in temperate regions limiting survivability, thereby constraining overall distribution.

The influence of rice, wind speed, and topographic features on the two pest species has not been effectively evaluated in previous studies. The present study addressed this gap by including all three variables in the Maxent model. Results revealed that the contribution of these variables to the model was lower than that of bioclimatic variables (Figure 3), indicating that bioclimate is the predominant determinant of *N. lugens and S. furcifera* distribution at the macroscale. Notably, macroscale relationships between pest occurrence and rice‐growing area may be weakened in the model, reducing the contribution of this variable. The model also showed that rice‐growing area has a stronger influence on *N. lugens* than on *S. furcifera* , likely because *N. lugens* feeds exclusively on rice (Heong et al. 2015), making its distribution highly dependent on rice availability. In contrast, *S. furcifera* also feeds on other crops, such as wheat and corn (Heong et al. 2015), diminishing the independent importance of rice‐growing area in its distribution. Both *N. lugens* and *S. furcifera* are small‐bodied insects with severely limited flight capabilities (Chen et al. 1984; Hu et al. 2007; Wu et al. 2022), and their long‐distance migration depends largely on wind currents rather than active flight (Lu et al. 2017; Wu et al. 2022). The relatively low importance of wind speed in the present model suggests that wind‐assisted migration is not a major limiting factor; instead, the habitat's bioclimatic conditions play a more decisive role. Prior field investigations have reported occurrences of *N. lugens* and *S. furcifera* at elevations above 1800 m (Yin et al. 2017; Bao et al. 2018), indicating that topography is a weak limiting factor, consistent with the outcomes of the present model (Figure 3).

Although the prediction results of the Maxent model showed that the core suitability areas of both pests are located in Asia, there are notable dissimilarities in their overall distribution ranges, which are closely related to their different tolerances to key environmental variables. The response curves for BIO18 and BIO13 revealed that *S. furcifera* has a lower precipitation limit than *N. lugens* (Table 1), indicating that the former requires less moisture and consequently exhibits greater drought tolerance, enabling it to survive in relatively arid environments. Additionally, the response curve for BIO6 demonstrated that the minimum suitable temperature for *S. furcifera* (−19.56°C) is lower than that for *N. lugens* (−15.01°C). This cold tolerance characteristic enables *S. furcifera* to establish larger populations in colder regions, such as Europe (Figure 5). In summary, the stronger tolerance of *S. furcifera* to both low temperature and high drought conditions allows it to occupy a broader distribution range.

Compared with previous studies predicting suitability areas for *N. lugens* and *S. furcifera* (e.g., Xiu et al. 2024; Surmaini et al. 2024), a major benefit of the present study is the development of a global Maxent model, enabling identification of suitable habitat patterns worldwide. Asia, which has the largest rice‐growing area and production globally, relies heavily on rice as a staple food. Notably, the current model predicts that Asia, especially China, contains the largest total and high suitability areas for *N. lugens* and *S. furcifera* (Figure 5), underscoring the serious threat these pests pose to the world's major rice‐producing regions and highlighting the need for strengthened pest management strategies. Additionally, the predicted suitability areas for these pests exceed the geographical scope of known occurrence records. For example, regions such as Brazil and Argentina are projected to be suitable habitats, although no presence has been recorded in these areas to date. One possible explanation is that population densities in these areas are currently excessively low to be detected.

Model predictions indicated that suitability areas for *N. lugens* and *S. furcifera* are likely to expand and shift northward under future climate scenarios, aligning with similar trends observed for other pest species, such as *Loxostege sticticalis* (Zhang et al. 2024), *Lycorma delicatula* (Zhao, Yang, and Chen 2024a), and *Metcalfa pruinose* (Zhao, Yang, Long, et al. 2024). These findings suggest that many high‐latitude regions are transitioning from unsuitable to suitable habitats for pest survival and population establishment. This also implies that rice‐growing areas vulnerable to infestation by *N. lugens* and *S. furcifera* may increase. To mitigate these emerging risks, appropriate pest management strategies must be developed for minimizing potential impacts on rice. Moreover, the developed model predicts an increase in high suitability areas for two pest species relative to the present period, indicating that the geographic range of pest outbreaks is likely to expand.

The predicted suitability areas identified in this study can inform targeted pest management planning. Asia remains the core region of infestation for *N. lugens* and *S. furcifera* , where control measures, such as pesticide applications, use of natural enemies, and deployment of resistant rice varieties, should be prioritized (Wang et al. 2017; Iamba and Dono 2021; Reddy et al. 2022). Additionally, population monitoring systems should be established in high suitability areas, such as China, to track pest population dynamics in real time. If abnormal population growth is detected, rapid response measures should be implemented to prevent severe crop losses. In newly suitable or expanding regions, strict quarantine protocols are necessary to prevent pest invasions. In areas where the pests are consistently present, long‐term and sustained management efforts are essential. Furthermore, because suitability areas span multiple countries, international cooperation is crucial for effective pest control. Countries should share data on population monitoring, outbreak‐associated environmental conditions, and successful control measures to enhance global prevention and response capabilities. Notably, a small portion of the suitability areas identified in this study is located within extrapolation zones. Caution should be exercised when applying these results to pest management as the associated predictions were generated under novel environmental conditions. However, these suitability areas are not invalid; they are simply less reliable. Future validation using other species distribution models could be conducted to further confirm them.

## 5 Conclusions

This study predicted the current and future global suitability areas for *N. lugens* and *S. furcifera* using a Maxent model. The model demonstrated that precipitation‐ and temperature‐related variables are the key limiting factors shaping their distributions. Results showed that current and future suitability areas are mainly concentrated in Asia and will expand and shift northward under climate change. Furthermore, the predicted suitability areas are expected to inform the development of management strategies, such as monitoring systems and strict quarantine protocols, to ensure effective and timely control of these agricultural pests.

## Author Contributions

**Zhengxue Zhao:** conceptualization (lead), data curation (lead), formal analysis (lead), funding acquisition (supporting), writing – original draft (lead). **Xueli Feng:** data curation (supporting), visualization (supporting). **Yingjian Wang:** funding acquisition (supporting). **Yubo Zhang:** funding acquisition (supporting), methodology (lead), resources (supporting).

## Funding

This work was supported by Guizhou Provincial Science and Technology Projects (QKHJC[2024]youth290 and QKHJCMS[2025]075), Scientific Research Project of Education Department of Guizhou Province (Qianjiaoji [2022] 326), and the Scientific Research Platform of the Education Department of Guizhou Province (Qianjiaoji[2022]052).

## Conflicts of Interest

The authors declare no conflicts of interest.

## Data Availability

The species occurrence records and R code are provided in figshare: https://doi.org/10.6084/m9.figshare.30630131.
